# Comparison of clinical and MRI features of brain metastases between ALK+ and ALK‐ NSCLC

**DOI:** 10.1002/cam4.7405

**Published:** 2024-06-17

**Authors:** Xiaolu Ren, Xuting Zhang, Xiaoyan Lei, Weiqin Ma, Ting Zhang, Yuxiang Wang, Jiwei Ren

**Affiliations:** ^1^ Department of Radiotherapy Shanxi Province Cancer Hospital/Shanxi Hospital Affiliated to Cancer Hospital, Chinese Academy of Medical Sciences/Cancer Hospital Affiliated to Shanxi Medical University Taiyuan Shanxi China; ^2^ Department of Radiology Shanxi Province Cancer Hospital/Shanxi Hospital Affiliated to Cancer Hospital, Chinese Academy of Medical Sciences/Cancer Hospital Affiliated to Shanxi Medical University Taiyuan Shanxi China; ^3^ Institute of Medical Imaging Shanxi Medical University Taiyuan Shanxi China; ^4^ Department of Ultrasound Shanxi Province Cancer Hospital/Shanxi Hospital Affiliated to Cancer Hospital, Chinese Academy of Medical Sciences/Cancer Hospital Affiliated to Shanxi Medical University Taiyuan Shanxi China

**Keywords:** anaplastic lymphoma kinase, brain metastases, MRI, nomogram, NSCLC, radiotherapy

## Abstract

**Background:**

Non‐small‐cell lung cancer (NSCLC) is the primary cause of brain metastases (BM). This study aimed to investigate differences in clinical and magnetic resonance imaging (MRI) features of BM between anaplastic lymphoma kinase (ALK) gene fusion (ALK+) and ALK wild‐type (ALK‐) NSCLC, and to preliminarily assess the efficacy of radiotherapy for treating BM.

**Methods:**

A retrospective analysis included 101 epidermal growth factor receptor (EGFR)‐ NSCLC patients with BM: 41 with ALK gene fusion and 60 being ALK‐. The brain MRI and clinical features were compared between different ALK status using the multivariate analysis, and a nomogram was constructed to predict ALK gene fusion. Fifty‐six patients who did not undergo cerebral surgery and had complete pre‐ and post‐ treatment data were further divided based on whether they received radiotherapy. Log‐rank test was used to compare the short‐term effect of treatment between the two groups under different genotypes.

**Results:**

ALK+ BM exhibited decreased peritumoral brain edema size, lower peritumoral brain edema index (PBEI), and a more homogeneous contrast enhancement pattern compared to ALK‐ BM. Age (OR = 1.04; 95%CI: 1.02–1.06), time to BM (OR = 1.50; 95% CI: 1.04–2.14), PBEI (OR = 1.26; 95% CI: 0.97–1.62), smoking status (smoking index >400 vs. non‐smoking status: OR = 1.42; 95% CI: 0.99–2.04) and contrast enhancement pattern (OR = 1.89; 95% CI: 1.28–2.78) were associated with ALK gene fusion. A nomogram based on these variables demonstrated acceptable predictive efficiency (AUC = 0.844). In the ALK+ group, patients who received radiotherapy did not show increased disease control rate (DCR) or progression‐free survival (PFS). In contrast, in the ALK‐ group, those who received radiotherapy had improved objective response rate (ORR), DCR, and PFS compared to those who were only treated with systemic therapy.

**Conclusions:**

The clinical and MRI features of BM can indicate the status of ALK in NSCLC. In the ALK‐ group, patients who received radiotherapy showed higher ORR, DCR, and PFS compared to those who did not.

## 1 BACKGROUND

Non‐small‐cell lung cancer (NSCLC) is the primary cause of brain metastases (BM),^1^ with 7%–10% of NSCLC patients presenting with BM at diagnosis and 20%–40% during treatment.^2^ Recent advancements in treatment strategies have extended NSCLC patient survival, consequently increasing the incidence of BM.^3^ Genetic testing in clinical settings is often hampered by insufficient biopsy samples, limiting secondary biopsies. Liquid biopsy, while a viable alternative, depends on adequate circulating tumor DNA, which may not fully align with tumor tissue genotypes, leading to potential inaccuracies.

Therefore, combining clinical assessment, imaging, and liquid biopsy is critical for accurate genotypic evaluation. Several studies have indicated that magnetic resonance imaging (MRI) parameters can somewhat predict epidermal growth factor receptor (EGFR) status.^4^, ^5^, ^6^, ^7^ However, limited research has focused on the correlation between anaplastic lymphoma kinase (ALK) status and brain MRI features. One study^8^ compared MRI features of BM in ALK‐positive and EGFR‐positive lung cancer patients and revealed that ALK‐positive patients were less likely to develop leptomeningeal spread. Nonetheless, the small sample size and incomplete data limit the generalizability of these findings.

Additionally, the efficacy of radiotherapy on BM in NSCLC patients with ALK gene fusions has not been determined.^9^, ^10^, ^11^, ^12^ This study aimed to assess ALK status using brain MRI features and to analyze the efficacy of intracranial radiotherapy in NSCLC patients with an ALK gene fusion.

## 2 METHODS

This study was approved by our Research Ethics Committee (No. 201835). Given the retrospective nature of the study, the requirement for informed consent was waived.

### 2.1 Patients

We retrospectively reviewed NSCLC patients treated and genetically tested at Shanxi Province Cancer Hospital from January 2013 to December 2021. The study included 147 patients with ALK+/EGFR− tumors without prior anti‐tumor therapy. ALK− gene fusion and EGFR gene mutations in primary lung tissues were detected using the amplification refractory mutation system (ARMS) on a real‐time automatic medical PCR analysis system. Brain MRI was primarily conducted on a 1.5 T Siemens scanner prior to any treatment. Among these patients, 49 with BM were selected. For comparison, 383 ALK−/EGFR− patients who received treatment during the same period at our hospital were randomly chosen, 71 of whom had BM.

Inclusion criteria were pathologically confirmed NSCLC, ALK, and EGFR gene detection, brain MRI–confirmed BM prior to treatment, and complete clinical/imaging data. Exclusion criteria were EGFR mutation, history of other tumors, and poor MRI image quality. Finally, a total of 101 patients who met the criteria were included in the analysis (41 ALK+ and 60 ALK−).

Data on age, gender, smoking status, time to BM, neurological symptoms, lung tumor pathology, tumor location, intracranial intervention were recorded. Time to BM was classified as synchronous (≤6 months from NSCLC diagnosis) or metachronous (>6 months).

For patients without intracranial surgery, patients who received the whole‐brain or focal radiotherapy were included in the radiotherapy group, while the non‐radiotherapy group consisted of patients treated with chemotherapy, targeted therapy, or immunotherapy excluding radiotherapy. Their progression‐free survival (PFS), defined as the time from radiotherapy completion to intracranial disease progression or the last follow‐up, were calculated and posttreatment brain MRI features were analyzed.

### 2.2 MRI features of BM

**FIGURE 1:**
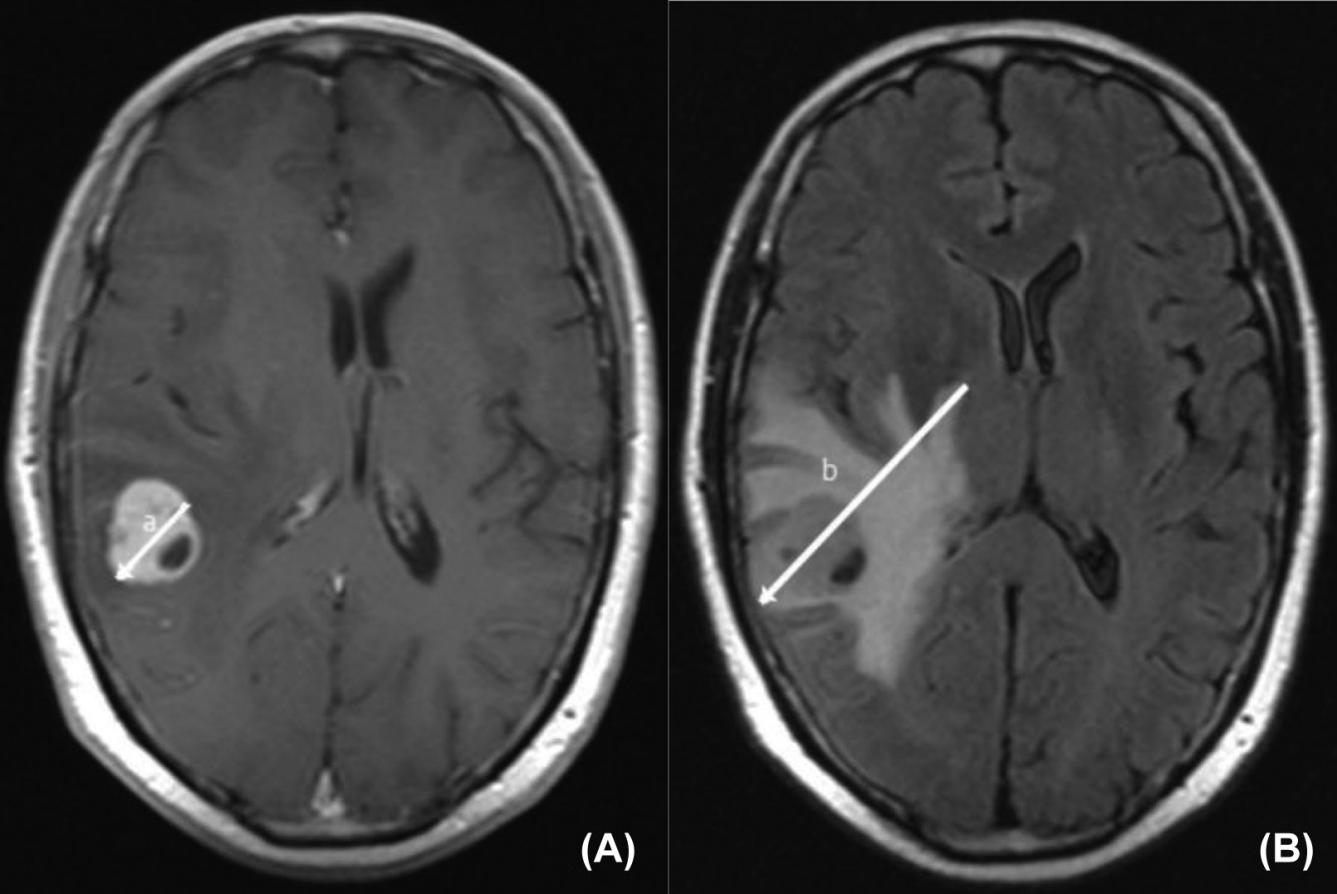
Method of measuring brain metastasis. (A) The maximum diameter of the tumor was measured via T1‐weighted Gd‐enhanced imaging (unidirectional arrow labeled “a”); (B) Peritumoral brain edema size was measured via T2‐FLAIR (unidirectional arrow labeled “b”).

Baseline brain MRI features were analyzed for the following variables: (I) number of BM; (II) size of the largest BM (on T1‐weighted imaging); (III) peritumoral brain edema size (PTBE), calculated as the difference between diameters on T1‐weighted Gd‐enhanced and T2‐FLAIR imaging; (IV) distribution of BM across various brain regions; (V) contrast enhancement patterns; (VI) presence of hemorrhage or necrosis. The peritumoral brain edema index (PBEI), as proposed by Chu^13^ and Tung,^14^ was calculated by dividing PTBE by tumor size. All measurements were conducted by two experienced radiologists blinded to clinical and ALK status (Figure 1).

### 2.3 Evaluation of radiotherapy efficacy

We evaluated the efficacy of radiotherapy on BM in 56 patients using posttreatment brain MRI. Treatment responses were categorized as per RECIST 1.1 criteria into complete remission (CR), partial remission (PR), stable disease (SD), and progressive disease (PD). The objective response rate (ORR) was calculated as the percentage of patients who achieved CR or PR, while the disease control rate (DCR) included patients who achieved CR or PR and those with SD.

### 2.4 Statistical analysis

Statistical analyses were conducted using the R programming language. Categorical variables were tested using the chi‐square test or Fisher's exact test, while continuous variables were analyzed using the *t*‐test or rank‐sum test. Variables with a *p* < 0.05 in the univariate analysis were included in the multivariate logistic regression. The final variables, chosen based on the minimum Akaike information criterion, were used to construct a nomogram for predicting ALK gene fusion. The nomogram's predictive efficiency was evaluated by receiver operating characteristic curve analysis, the area under the curve (AUC) was calculated, and calibration curve and decision curve analyses (DCAs) were performed. The AUC values were also validated through five‐fold cross‐validation. The ORR and DCR differences between radiotherapy and non‐radiotherapy groups were compared using the chi‐square test. Survival curves were plotted using the Kaplan–Meier method, with PFS differences assessed using the log‐rank test.

## 3 RESULTS

### 3.1 Baseline characteristics

The study included 101 BM patients, 41 with ALK+ tumors and 60 with ALK‐ tumors. The cohort comprised 69 males and 32 females, with a median age of 61 years (Table 1). Most patients (96) had adenocarcinoma, while five had other NSCLC subtypes. Thirty‐seven patients had more than three BM, while 64 had three or fewer BM.

**TABLE 1:** Clinical characteristics of patients.

Characteristic	ALK+ group (*n* = 41)	ALK− group (*n* = 60)	*p*‐value
Age, years, median (IQR)	54 (50, 61)	63 (56, 67)	<0.001
Gender, *n* (%)	–	–	0.005
Male	21 (51.2)	48 (80.0)	–
Female	20 (48.8)	12 (20.0)	–
Smoking status, *n* (%)	–	–	0.001
No smoking	30 (73.2)	22 (36.7)	–
Smoking index ≤400	2 (4.9)	6 (10.0)	–
Smoking index >400	9 (22.0)	32 (53.3)	–
Neurological symptoms, *n* (%)	–	–	0.100
Yes	15 (35.7)	38 (63.3)	–
No	24 (57.1)	22 (36.7)	–
Unknown	3 (7.1)	0 (0.0)	–
Pathology of lung cancer, n (%)	–	–	1.000
Adenocarcinoma	39 (95.1)	57 (95.0)	–
Others	2 (4.9)	3 (5.0)	–
Site of the lesion, n (%)	–	–	0.471
Right lobe	16 (39.0)	29 (48.3)	–
Left lobe	25 (61.0)	31 (51.7)	–
Time to BM, *n* (%)	–	–	0.005
Metachronous (>6 months)	19 (46.3)	11 (18.3)	–
Synchronous (≤6 months)	22 (53.7)	49 (81.7)	–

Abbreviations: ALK, anaplastic lymphoma kinase; BM, brain metastasis.

### 3.2 Construction of the nomogram

**FIGURE 2:**
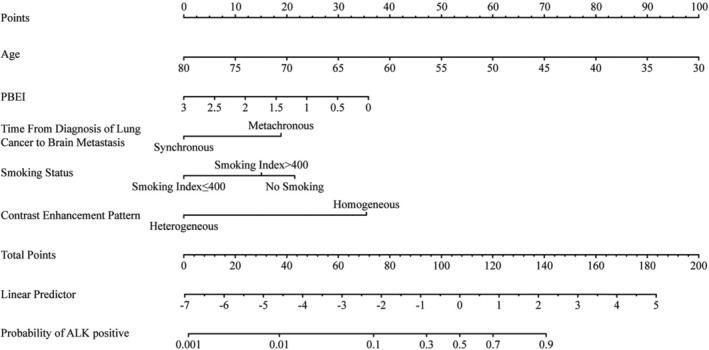
A nomogram was constructed to predict anaplastic lymphoma kinase (ALK) gene status. The nomogram included age, time to brain metastases (BM), smoking status, contrast enhancement pattern, and peritumoral brain edema index. The time from diagnosis of lung cancer to detection of BM was considered the time to BM.

Univariate analysis indicated that the ALK+ group had a younger median age than ALK− patients (54 [50, 61] vs. 63[56, 67] years, *p* < 0.001), more females (48.8% vs. 20%, *p* < 0.005), and more non‐smokers (73.2% vs. 36.7%, *p* < 0.001) (Table 1). MRI characteristics distinguishing the ALK+ group presented with smaller PBEI (median: 0.08 vs. 0.46, *p* < 0.05), smaller peripheral brain edema size (PBES, median: 0.1 cm vs. 1 cm, *p* < 0.05), more homogeneous enhancement (34.1% vs. 13.3%, *p* < 0.05), and a higher rate of metachronous time to BM (46.3% vs. 18.3%, *p* < 0.01) (Table 2). The other characteristics showed no significant difference (all *p* > 0.05). Multivariate logistic regression identified age, time to BM, PBEI, smoking status, and contrast enhancement pattern as significant factors correlated to ALK status. The resultant nomogram was developed based on these variables (Table 3; Figure 2).

**TABLE 2:** MRI features of the brain metastases.

Characteristic	ALK+ (*n* = 41)	ALK− (*n* = 60)	*p*‐value
BM size, cm, median (IQR)	1.60 (0.80, 4.90)	2.55 (1.10, 5.30)	0.134
BM numbers, *n* (%)			0.827
≤3	27 (65.9)	37 (61.7)	
>3	14 (34.1)	23 (38.3)	
BM location, *n* (%)			0.225
Above the curtain	21 (51.2)	33 (55.0)	
Under the curtain	5 (12.2)	2 (3.3)	
Above and under the curtain	15 (36.6)	25 (41.7)	
PBEI, median (IQR)	0.08 (0.00, 0.57)	0.46 (0.28, 0.96)	0.010
PBES, median (IQR)	0.10 (0.00, 1.70)	1.00 (0.38, 3.02)	0.016
Contrast enhancement pattern, *n* (%)			0.025
Homogeneous	14 (34.1)	8 (13.3)	
Heterogeneous	27 (65.9)	52 (86.7)	
Necrosis, *n* (%)			1.000
Yes	2 (4.9)	3 (5.0)	
No	39 (95.1)	57 (95.0)	
Hemorrhage, *n* (%)			1.000
Yes	17 (41.5)	26 (43.3)	
No	24 (58.5)	34 (56.7)	
Form, *n* (%)			0.390
Nodular type	34 (82.9)	48 (80.0)	
Irregular type	6 (14.6)	12 (20.0)	

Abbreviations: BM, brain metastasis; MRI, magnetic resonance imaging; PBEI, peritumoral brain edema index; PBES, peritumoral brain edema size.

**TABLE 3:** Factors related to ALK status according to multivariate logistic regression analysis.

Characteristic	Odds ratio (95% CI)	*p*‐value
Time to BM (metachronous, synchronous)	1.494 (1.041–2.143)	0.032
Age	1.036 (1.017–1.055)	0.001
PBEI	1.255 (0.973–1.618)	0.083
Smoking status		
Smoking index ≤400 versus no smoking	1.590 (0.861–2.938)	0.142
Smoking index >400 versus no smoking	1.418 (0.987–2.037)	0.062
Contrast enhancement pattern		
Heterogeneous versus homogeneous	1.888 (1.280–2.783)	0.002

Abbreviations: ALK, anaplastic lymphoma kinase; BM, brain metastasis; PBEI, peritumoral brain edema index.

### 3.3 Verification of the nomogram

**FIGURE 3:**
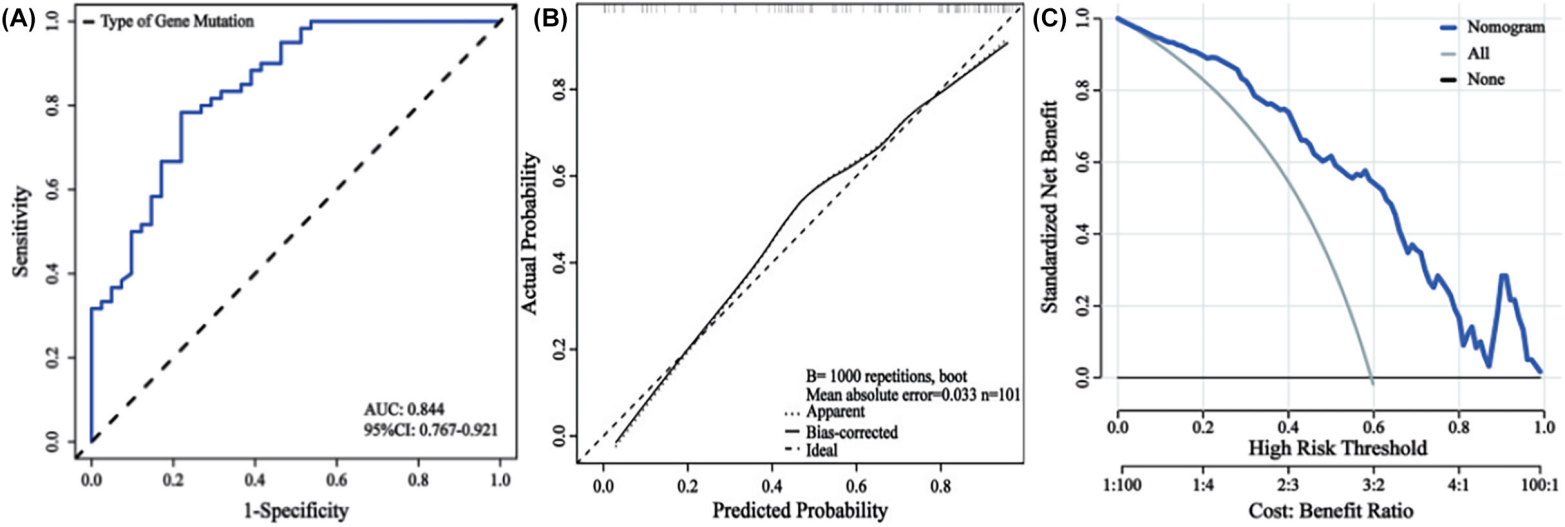
Verification of the nomogram. (A) The receiver operating characteristic (ROC) curve: the area under the curve was 0.844 (95% CI: 0.767–0.921). (B) Calibration curve: mean absolute error = 0.033. (C) Decision curve analysis (DCA) demonstrated greater clinical efficacy at a threshold probability of 0.01–0.99.w

The nomogram demonstrated high discriminative ability (AUC: 0.844; 95% CI: 0.767–0.921) and accurate calibration. DCA affirmed its clinical utility across a broad probability threshold (0.01–0.99). The nomogram's repeatability was confirmed via five‐fold cross‐validation (average AUC values: 0.843 and 0.828) (Table 4; Figure 3).

**TABLE 4:** The AUC values of five‐fold cross validation.

	Minimum	Q25	Median	Average	Q75	Maximum
Training set	0.824	0.829	0.842	0.843	0.858	0.862
Test set	0.763	0.774	0.859	0.828	0.870	0.875

Abbreviation: AUC, area under the curve.

### 3.4 Analysis of the curative effect of radiotherapy

Among all patients, 55 patients underwent intracranial surgery, 25 received radiotherapy for BM, and 31 had no intracranial intervention. This analysis focused on 56 patients who had complete follow‐up data and had not undergone surgery, including 25 who received brain radiotherapy (the radiotherapy group) and 31 who did not (the non‐radiotherapy group). Of them, 21 were ALK+ (7 in the radiotherapy group and 14 in the non‐radiotherapy group) and the remaining were ALK−. General characteristics of these groups are detailed in Table 5. The median PFS for all 56 evaluated patients was 9.8 months (95% CI: 0.8 to 37.1).

**TABLE 5:** Clinical characteristics of patients receiving radiation.

Characteristic	RT (*n* = 25)	Non‐RT (*n* = 31)	*p*‐value
Age, years, median (IQR)	63 (52, 67)	56 (52, 63)	0.195
ALK+, *n* (%)			0.187
Yes	7 (28.0)	14 (45.0)	
No	18 (72.0)	17 (54.8)	
Gender, *n* (%)	–	–	0.015
Male	20 (80.0)	15 (48.4)	–
Female	5 (20.0)	16 (51.6)	–
Smoking status, *n* (%)	–	–	0.501
No smoking	13 (52.0)	20 (64.5)	–
Smoking index ≤400	2 (8.0)	1 (3.2)	–
Smoking index >400	10 (40.0)	10 (32.3)	–
Neurological symptoms, *n* (%)	–	–	0.011
Yes	14 (56.0)	6 (19.4)	–
No	11 (44.0)	24 (77.4)	–
Unknown	0 (0.0)	1 (3.2)	–
Pathology of lung cancer, n (%)	–	–	0.848
Adenocarcinoma	23 (92.0)	30 (96.8)	–
Others	2 (8.0)	1 (3.2)	–
Site of the primary lesion, *n* (%)	–	–	0.295
Right lobe	11 (44.0)	18 (58.1)	–
Left lobe	14 (56.0)	13 (41.9)	–
Time to BM, *n* (%)	–	–	0.400
Metachronous (>6 months)	7 (28.0)	12 (38.7)	–
Synchronous (≤6 months)	18 (72.0)	19 (61.3)	–

Abbreviations: ALK, anaplastic lymphoma kinase; BM, brain metastasis; non‐RT, non‐radiotherapy group; RT, radiotherapy group.

**FIGURE 4:**
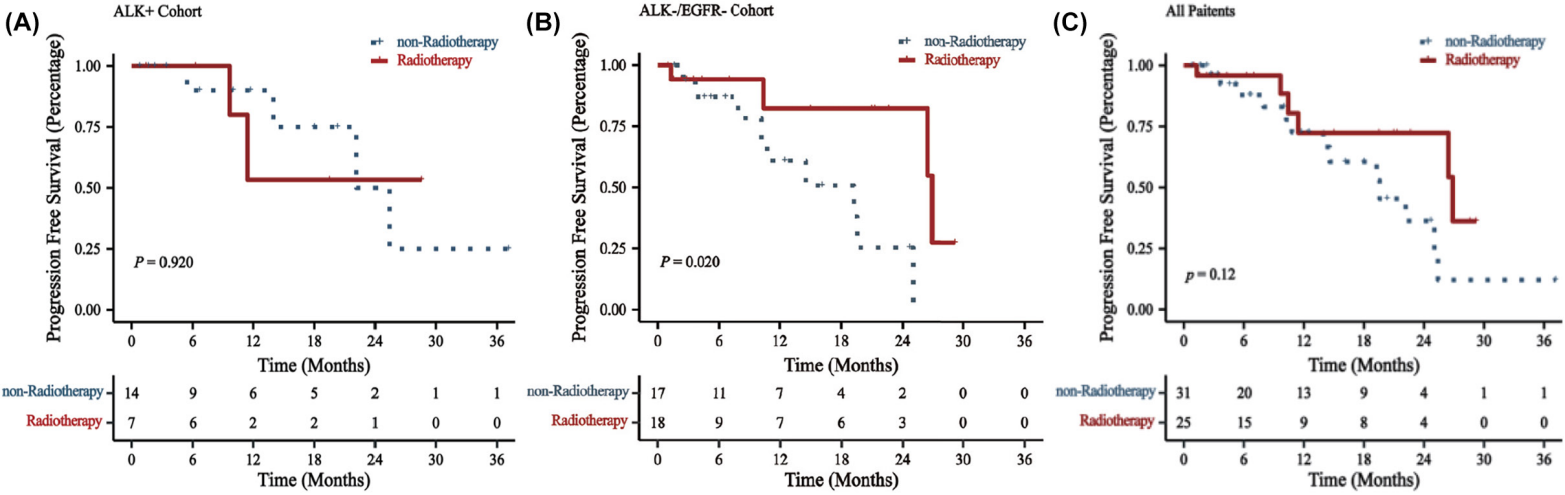
Kaplan–Meier survival curves for patients who received intracranial radiotherapy (continuous line) versus patients whom not (dotted line). (A) ALK+ group: *p* = 0.92; (B) ALK− group: *p* = 0.02; (C) all patients: *p* = 0.12.

Among the ALK+ patients, the radiotherapy group exhibited a lower ORR compared to the non‐radiotherapy group (*p* < 0.05), with no significant differences in DCR and PFS (*p* > 0.05). In ALK− patients, the radiotherapy group had a greater ORR and DCR (*p* < 0.05) and better PFS (*p* < 0.05), than those of the non‐radiotherapy group. Among all 56 patients, the radiotherapy group had a higher DCR (*p* < 0.05), with no notable differences in ORR and PFS (*p* > 0.05) (Table 6; Figure 4).

**TABLE 6:** Analysis of the curative effect of radiotherapy.

		*N*	CR (*n*)	PR (*n*)	SD (*n*)	PD (*n*)	ORR (%)	DCR (%)
ALK+	RT	7	1	1	3	2	28.600	71.400
	non‐RT	14	4	3	3	4	50.000	71.400
	*X* ^2^						9.599	0.000
	*p*‐value						0.002	1.000
ALK‐	RT	18	1	9	4	4	55.600	77.800
	non‐RT	17	2	3	3	9	29.400	47.100
	*X* ^2^						14.045	20.096
	*p*‐value						<0.001	<0.001
Total	RT	25	2	10	7	6	48.000	76.000
	non‐RT	31	6	6	6	13	38.700	58.100
	*X* ^2^						1.761	7.251
	*p*‐value						0.185	0.007

Abbreviations: ALK, anaplastic lymphoma kinase; CR, complete remission; DCR, disease control rate; ORR, objective response rate; PD, progressive disease; PR, partial remission; RT, radiotherapy group; SD, stable disease; non‐RT, non‐radiotherapy group.

## 4 DISCUSSION

This study offers an extensive analysis of brain MRI features in NSCLC patients, particularly examining differences between the ALK+ and ALK− groups. We established that age, smoking index, PBEI, contrast enhancement pattern, and time to BM are significantly associated with ALK status, leading to the creation of a predictive model for ALK gene fusion. Our comparison of the curative efficacy of radiotherapy in the ALK+ and ALK− groups revealed that ALK+ patients exhibited a less favorable short‐term response to radiotherapy than the other patients, consistent with clinical observations.

We observed that ALK+ patients were commonly younger, female, non‐smokers, and had a greater incidence of metachronous BM. These findings align with those of Mendoza et al.,^15^ who reported similar demographic tendencies in ALK+ NSCLC patients and other existing literature.^16^, ^17^ The increased incidence of metachronous BM in the ALK+ group could be attributed to the longer survival duration of these patients.^18^, ^19^ Additionally, BM in ALK+ NSCLC patients were more commonly located in the right middle occipital gyrus, right posterior cingulate, right precuneus, right precentral gyrus, and right parietal lobe.^20^

Regarding the prediction of ALK status from brain MRI characteristics, there is no established consensus. T2‐FLAIR and T1‐CE radiomics were suggested to better identify EGFR and ALK mutation status compared to T1‐CE MR sequence.^21^ There was a case of ALK+ NSCLC with BM presented with MRI features resembling an abscess.^22^ Ullas et al.,^8^ observed more central necrosis in the ALK mutation group compared to the EGFR mutation group in BM (8/24 vs 2/68). Inconsistent with these findings, our results revealed no significant association between necrosis and ALK gene fusion in comparison to that in the ALK− group (17/41 vs. 26/60, *p* > 0.05). Moreover, we noted a decreased PBEI and PBES in the ALK+ group (*p* < 0.05), in contrast with the findings of Ullas et al.,^8^ who reported no significant differences in perilesional edema between the ALK and EGFR mutation groups.

To our knowledge, several studies suggest that MRI features can somewhat indicate EGFR status, with patients in the exon 19 deletion subgroup exhibiting less peritumoral brain edema than patients in the EGFR wild‐type subgroup.^4^ Our findings suggest that this difference may be related to the reduced invasiveness of gene‐mutant tumors. Additionally, we found a greater incidence of homogeneous density in BM in the ALK+ group. Currently, deep learning has been used to predict gene mutations based on MRI features. Abhishek's model found that ALK+ patients were more likely to presented with ring enhancing lesions than EGFR+ patients and had a higher probability of meningeal involvement compared to double negative groups.^23^ However, literature on this MRI feature remains scarce, preventing a clear consensus.

The study revealed no significant difference in DCR and PFS between the ALK+ group receiving radiotherapy and those not receiving it. However, for ALK− patients, the radiotherapy group showed higher ORR, DCR, and PFS compared to their non‐radiotherapy group. This suggests that intracranial radiotherapy may be less effective in the ALK+ group than in the ALK− group. For ALK+ NSCLC, Thomas^10^ also found no evident difference between TKI and CNS RT + TKI groups for the time to intracranial progression (18.1 vs. 21.8 months, *p* = 0.65). Moreover, in a retrospective study, for ALK–rearranged or EGFR–mutant lung cancer, Dutta et al.,^12^ reported that patients treated solely with TKIs achieved a 94% partial intracranial response at 3 months, whereas 58% of those receiving TKIs combined with radiation achieved this outcome. Correspondingly, ALK+ patients with BM often do not receive routine intracranial radiotherapy in clinical practice, possibly due to the superior intracranial control offered by ALK‐TKIs alone.^10^, ^11^, ^12^

This study has several limitations, including potential biases due to its small sample size, the low incidence of ALK+ tumors and the strict inclusion criteria. Given its retrospective nature, this study inherently lacks control over certain confounding variables. The gene mutation status, derived from primary lung cancer tissues, might not mirror secondary mutations in metastases. The single‐center nature of the study and lack of external validation also limit the general applicability of these findings. Future multi‐center studies and the integration of CT features of lung masses with brain MRI features are planned to enhance the use of the ALK gene fusion prediction model.

## 5 CONCLUSION

Significant differences in age, smoking index, PBEI, contrast enhancement pattern, and time to BM were detected between ALK+ and ALK− NSCLC patients with BM. An ALK gene fusion predictive model constructed from these variables provides valuable insights for histologically undetectable patients. In the ALK‐ group, patients who received radiotherapy had greater ORR, DCR, and PFS compared to those who did not.

## AUTHOR CONTRIBUTIONS

**Xiaolu Ren:** Conceptualization (equal); formal analysis (equal); investigation (equal); writing – original draft (equal). **Xuting Zhang:** Data curation (equal); formal analysis (equal); investigation (equal); writing – original draft (equal). **Xiaoyan Lei:** Investigation (supporting); writing – original draft (supporting). **Weiqin Ma:** Investigation (supporting). **Ting Zhang:** Investigation (supporting). **Yuxiang Wang:** Conceptualization (lead); supervision (lead); writing – review and editing (lead). **Jiwei Ren:** Conceptualization (lead); supervision (lead); writing – review and editing (lead).

## FUNDING INFORMATION

The current project was funded by the Science and Technology Commission of Shanxi Province (No. 202103021223447 and 202103021224407), the Science and Education Cultivation Fund of the National Cancer and Regional Medical Center of Shanxi Provincial Cancer Hospital (No.QH2023022).

## CONFLICT OF INTEREST STATEMENT

The authors declare that they have no competing interests.

## Data Availability

The datasets used and/or analyzed during the current study are available from the corresponding author on reasonable request.
